# Impact of Artificial Intelligence on Polyp Size and Surveillance Colonoscopy: A Phantom Study

**DOI:** 10.7759/cureus.74600

**Published:** 2024-11-27

**Authors:** Muhammad N Yousaf, Neal Sharma, Michelle L Matteson-Kome, Srinivas Puli, Douglas Nguyen, Matthew L Bechtold

**Affiliations:** 1 Department of Medicine/Gastroenterology and Hepatology, University of Missouri School of Medicine and University Hospital, Columbia, USA; 2 Department of Gastroenterology and Hepatology, Digestive Health Specialists, P.A., Winston-Salem, USA; 3 Department of Internal Medicine, Harry S. Truman Memorial Veterans' Hospital, Columbia, USA; 4 Department of Gastroenterology, University of Illinois at Peoria, Peoria, USA; 5 Division of Gastroenterology and Hepatology, Loma Linda University Medical Center, Loma Linda, USA; 6 Department of Gastroenterology, University of Missouri Columbia, Columbia, USA

**Keywords:** artificial intelligence, colonoscopy, endoscopy, polyps, surveillance

## Abstract

Background

Artificial intelligence (AI) is a hot topic in the world of medicine. AI may be useful in identifying and sizing polyps, which influence surveillance intervals. Therefore, we examined polyp size estimation by AI using a survey study.

Methods

A survey study was performed using a phantom colon model. Eleven videos were produced in the colon phantom using a colonoscope. Gastroenterologists were compared to a new AI system (Argus) for sizing polyps and their impact on surveillance intervals.

Results

Eleven gastroenterologists completed the survey with a mean age of 51.1 ± 8.1 years and an average of 19.3 ± 10 years of experience. Mean accuracy rates for gastroenterologists were 76% ± 0.1% (range 54-89%) compared to 96% ± 0.05% for Argus. Endoscopists estimated polyp size within ± 1 mm 44 times (36%) versus 9 times (82%) with Argus. Endoscopists' surveillance recommendations were significantly more often inappropriate compared to Argus (34 vs 0). The interval of next colonoscopy was too short for 27 endoscopists (22%) and too long for seven endoscopists (6%).

Conclusions

AI appears to be more accurate in estimating polyp size than experienced endoscopists. Given the potential impact on surveillance intervals, AI may result in cost savings.

## Introduction

Colorectal cancer (CRC) is a common cancer and a leading cause of cancer-related deaths in both men and women in the United States [1]. Risk factors for colon cancer include age (over 45), a personal or family history of colon cancer or polyps, a diet high in red or processed meats, obesity, and smoking [2-4]. Early detection of colon cancer is key to successful treatment and can improve outcomes for patients. There are different tests for colon cancer screening, including fecal occult blood testing (FOBT), fecal immunochemical testing (FIT), fecal DNA testing, and CT colonography; however, colonoscopy is considered one of the best tests for colon cancer screening [5,6].

A high-quality colonoscopy examination is a cost-effective approach for the detection and removal of premalignant polyps, avoiding the need for surgical resection of the majority of superficial submucosal polyps. A high-quality colonoscopy depends on the endoscopist’s experience, the quality of bowel preparation, and the utilization of innovative technology for the detection of colonic polyps. Current guidelines determine the interval of surveillance colonoscopy based on the number, size, and histology of colon polyps [2,3,7]. In current clinical practice, the size of polyps is determined by the endoscopist, and there is a tendency for variability in the sizing of polyps based on their experience. This variability in the calculation of polyp size may impact the interval of surveillance colonoscopy.

Artificial intelligence (AI) is an emerging technology increasingly utilized in all fields of medicine, including colonoscopy [8]. AI algorithms can analyze colonoscopy images and highlight areas of the colon that may contain polyps, helping endoscopists focus their attention on relevant areas and increasing the chances of detecting polyps that might otherwise be missed by human observers. This is particularly helpful in cases where the polyps are small or subtle, making them difficult to detect. In addition, AI algorithms can generate real-time patterns of colonoscopic images and videos, helping distinguish the probable histology of different types of polyps, such as hyperplastic polyps and adenomatous polyps, which have different risk profiles. AI-assisted colonoscopy has shown the potential to improve the polyp detection rate and increase the proportion of surveillance colonoscopies by 35% in the United States [9]. However, limited data are available comparing AI with endoscopists’ estimation of polyp size during colonoscopy. Wang P et al. demonstrated that an AI algorithm was more accurate than gastroenterologists in estimating the size of small polyps (less than 6 mm) [10]. However, in a double-blinded randomized study, the same group of authors showed that AI was comparable to gastroenterologists in estimating the size of larger polyps (6-10 mm) [11]. Standardization of polyp size estimation by endoscopists is necessary, as it may impact the interval of surveillance colonoscopy. AI could be a powerful tool for training endoscopists, helping them identify the correct polyp size, remove polyps more effectively, and determine the appropriate interval for follow-up colonoscopy, while minimizing the cost burden for patients and the healthcare system.

Due to the questionable impact of AI on colonoscopy, we performed a survey study comparing AI and experienced gastroenterologists for estimating polyp size and its impact on surveillance intervals.

## Materials and methods

This is a multicentered experimental study performed from May to June 2021. The study was approved by the institutional review board at the University of Missouri (project #2062726) as an exempt application, given that no identifiable patient health information was used. A scripted email was sent to experienced gastroenterologists asking them to participate in the study. If agreeable, a consent to participate letter was sent via email. Once consent was obtained, a group of experienced gastroenterologists voluntarily participated in this study.

Phantom model

**Figure 1:**
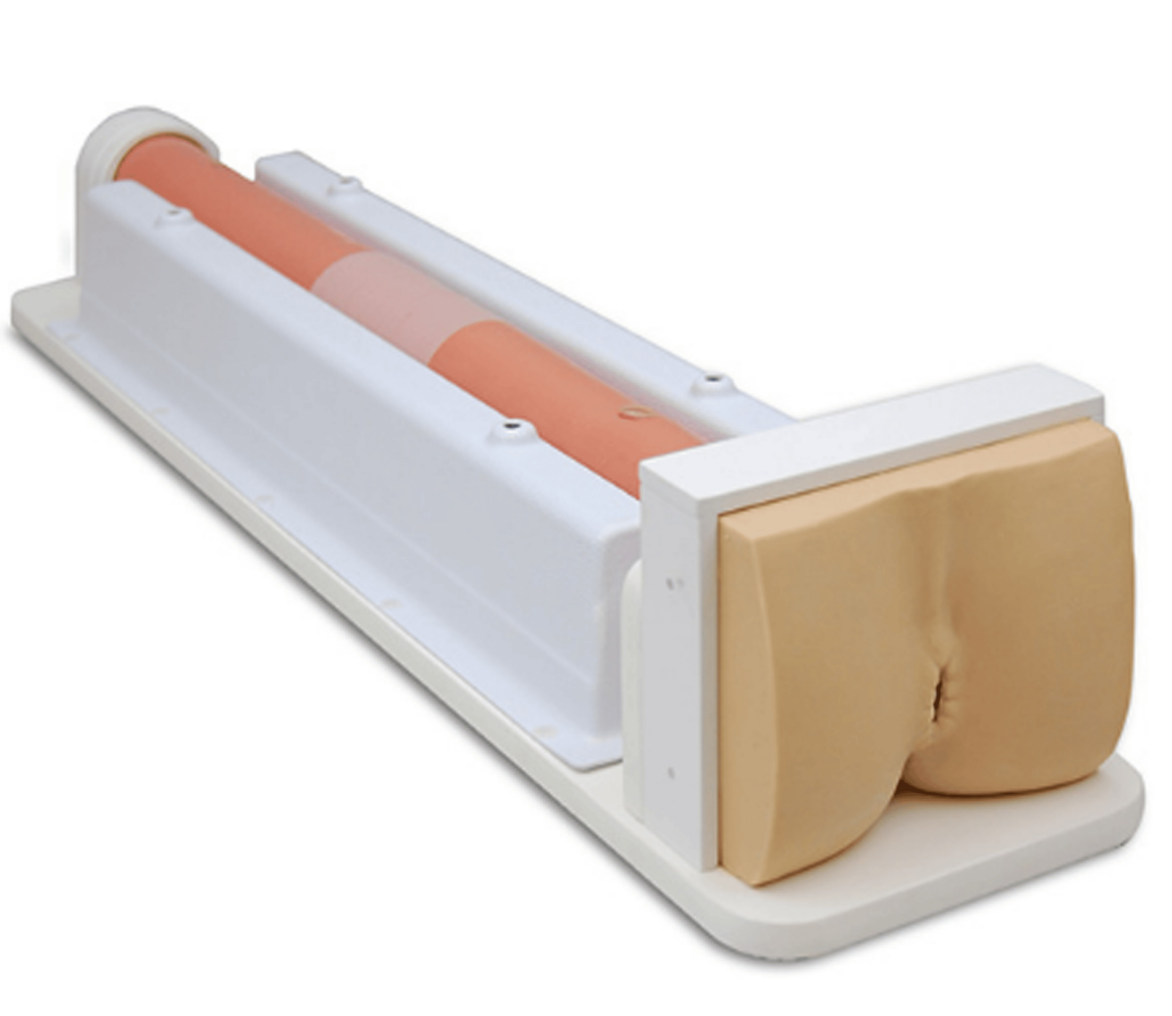
Colon phantom model. Image attributed to and permitted by The Chamberlain Group (34 Main Street, Great Barrington, MA 01230).

A phantom model of a colon with artificial colon polyps (rubber-based or modeling compound) was created by The Chamberlain Group (34 Main Street, Great Barrington, MA 01230) (Figure 1).

A digital caliper was used to size the artificially made polyps and to obtain the gold standard size.

**Figure 2:**
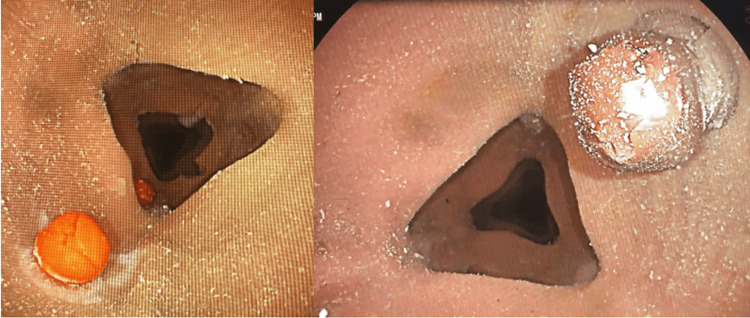
Polyps in the phantom model.

The polyps were labeled with numbers, and the size of each polyp was entered into the data collection spreadsheet. A total of 11 polyps were made, measured, and placed into the phantom colon model (Figure 2).

Polyp detection and sizing

In the first phase, a colonoscope was used on the phantom model to create eleven short videos for each polyp present in the colon phantom. Videos were provided by EndoSoft (EndoSoft LLC, Schenectady, NY 12305). After sending a letter of consent for participation in the study, these short eleven videos were sent to the endoscopists across the United States to visually estimate the size of the polyps as seen by the inserted scope. This step is identical to the way endoscopists visually estimate the size of polyps in a real colonoscopy procedure. The estimated sizes given by the endoscopists were registered in the spreadsheet, carrying the number of the “polyps” from 1 to 11 and their exact sizes measured with a digital caliper.

**Figure 3:**
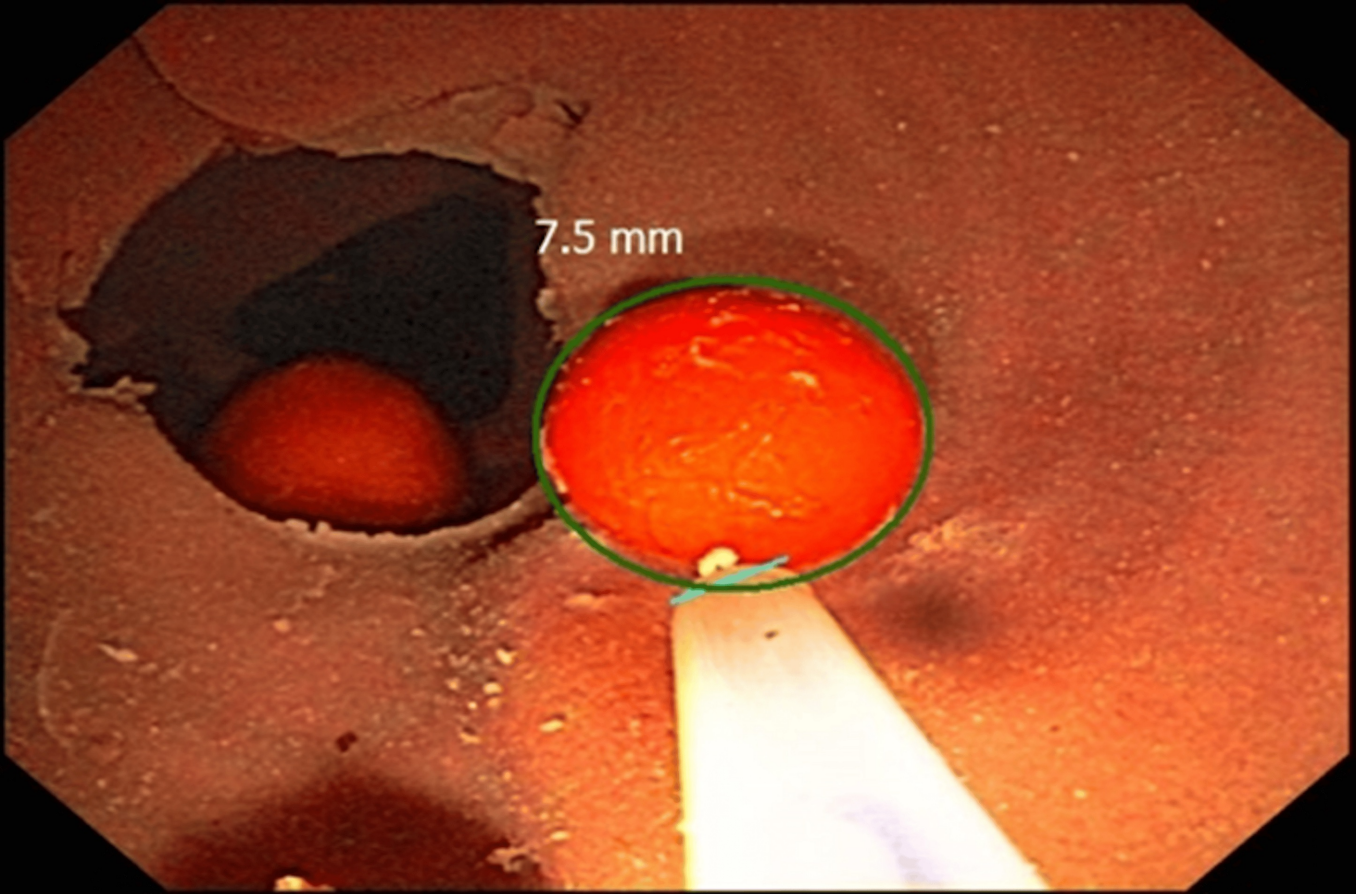
Argus measurement of a polyp in the phantom model, using a snare as a reference.

In the second phase, Argus (an AI technology software by EndoSoft - EndoSoft LLC, Schenectady, NY 12305) was used to detect the artificial polyp in the phantom model. With the assistance of a snare in proximity to the artificial polyp to serve as a sizing reference for the AI system, the size of the polyp was displayed on the AI monitor (Figure 3).

Data collection

We collected data for each polyp sized in the colon phantom model. The size of each polyp was taken with a digital caliper prior to the placement of the polyps in the phantom model. Polyp size measurements determined by Argus were collected using principles of deep learning and AI. A real-time video of colonoscopy on the phantom colon was recorded, and images of each polyp with the displayed size measurement were captured. In addition, we documented the number of instances when Argus failed to size the polyp. The endoscopists' visual estimation of each polyp size was also recorded from the review of 11 videos of each polyp recorded earlier.

Polyp size accuracy

The accuracy of polyp size in the phantom colon model between the endoscopists and the Argus AI system was measured by two methods. The first method used an accuracy rate on how close the measurement of the endoscopist or Argus was in comparison to the caliper measurement for each polyp, defined by a percentage. For example, if the polyp was 10 mm and the endoscopist or Argus measured the polyp at 8 mm, then the accuracy rate was determined to be 80%. The mean of this rate was calculated for each endoscopist and for Argus. The second method used accuracy as a categorical measurement. Accuracy was determined by the number of times the endoscopists or Argus were within ± 1 mm of the caliper measurement of the polyp.

Surveillance interval

The comparison of surveillance intervals was made between the endoscopists and Argus. All created polyps were assigned to be tubular adenomas with no high-grade dysplasia. The surveillance interval was based on the estimated size of the adenomatous polyp. For each polyp, the surveillance interval was determined as appropriate or inappropriate (too short or too long) for the endoscopists or Argus compared to the caliper measurement. The appropriate surveillance interval was based on the guidelines at the time of the study in 2021.

Statistical analysis

Results are presented as mean ± SD, range, or percentage (%). Demographic data were analyzed using descriptive statistics. Categorical data were analyzed using Fisher's exact test with significance defined as a p-value <0.05. Continuous data were analyzed using a t-test with significance defined as a p-value < 0.05.

## Results

A total of 11 artificial polyps were placed in the phantom colon with sizes ranging from 2.5 to 15 mm, measured with a digital caliper (Table 1).

**Table 1:** Details of artificial polyps inserted into the phantom model.

Polyp (#)	Size (mm)
1	9
2	2.5
3	5.5
4	6.5
5	8
6	15
7	9
8	8
9	8
10	6
11	10

**Figure 4:**
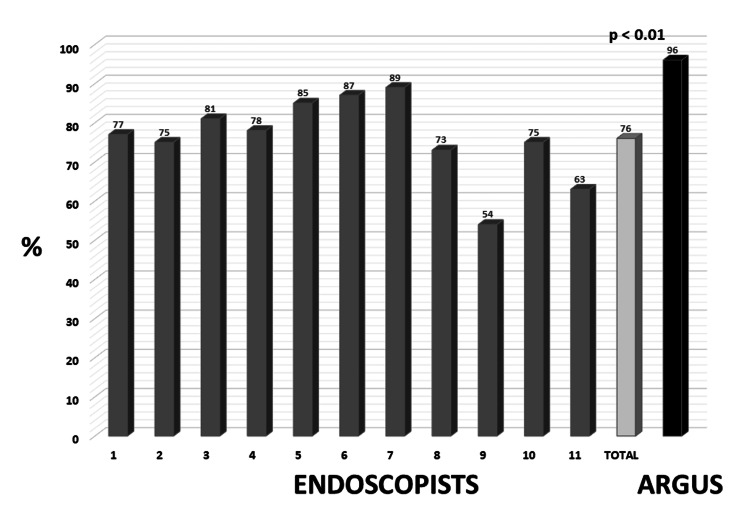
Mean accuracy rates for endoscopists compared to Argus.

Standardized videos were filmed for each polyp (n=11) with a high-definition colonoscope. The mean duration of the video clips was 13.9 ± 7.6 seconds (range 7-28 seconds). A group of 11 gastroenterologists from academic institutions across the United States volunteered and performed the survey. The mean age of the gastroenterologists was 51.1 ± 8.1 years, with a mean experience of 19.3 ± 10 years. The overall mean accuracy rates for gastroenterologists in estimating the exact polyp size were 76% ± 0.1% (range 54-89%) compared to 96% ± 0.05% for Argus (p < 0.01) (Figure 4).

**Figure 5:**
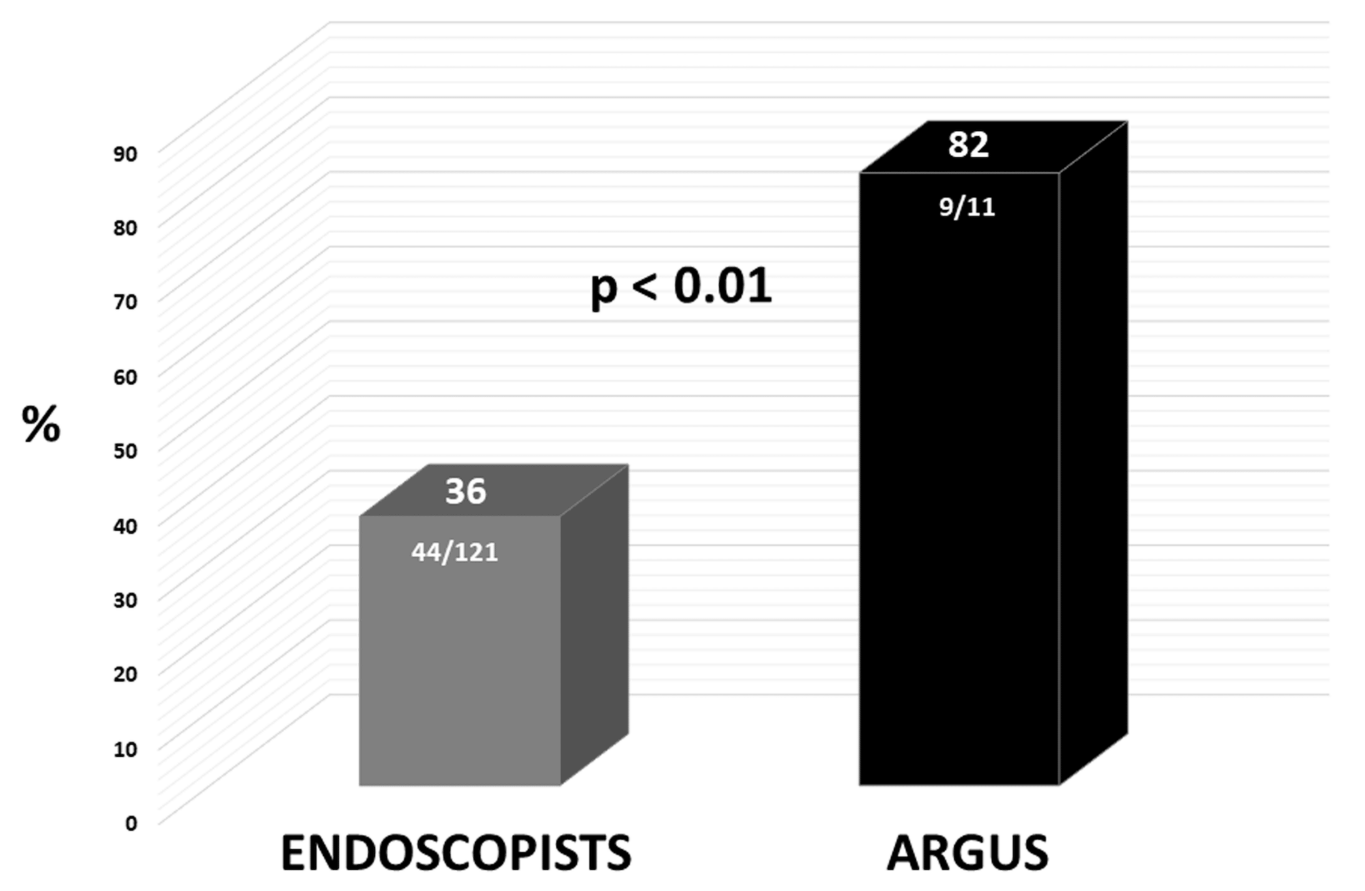
Accuracy within ≤ 1 mm for endoscopists and Argus.

 The endoscopists estimated polyp size within ± 1 mm 44 times (36%), while Argus did so 9 times (82%) (p < 0.01) (Figure 5).

**Figure 6:**
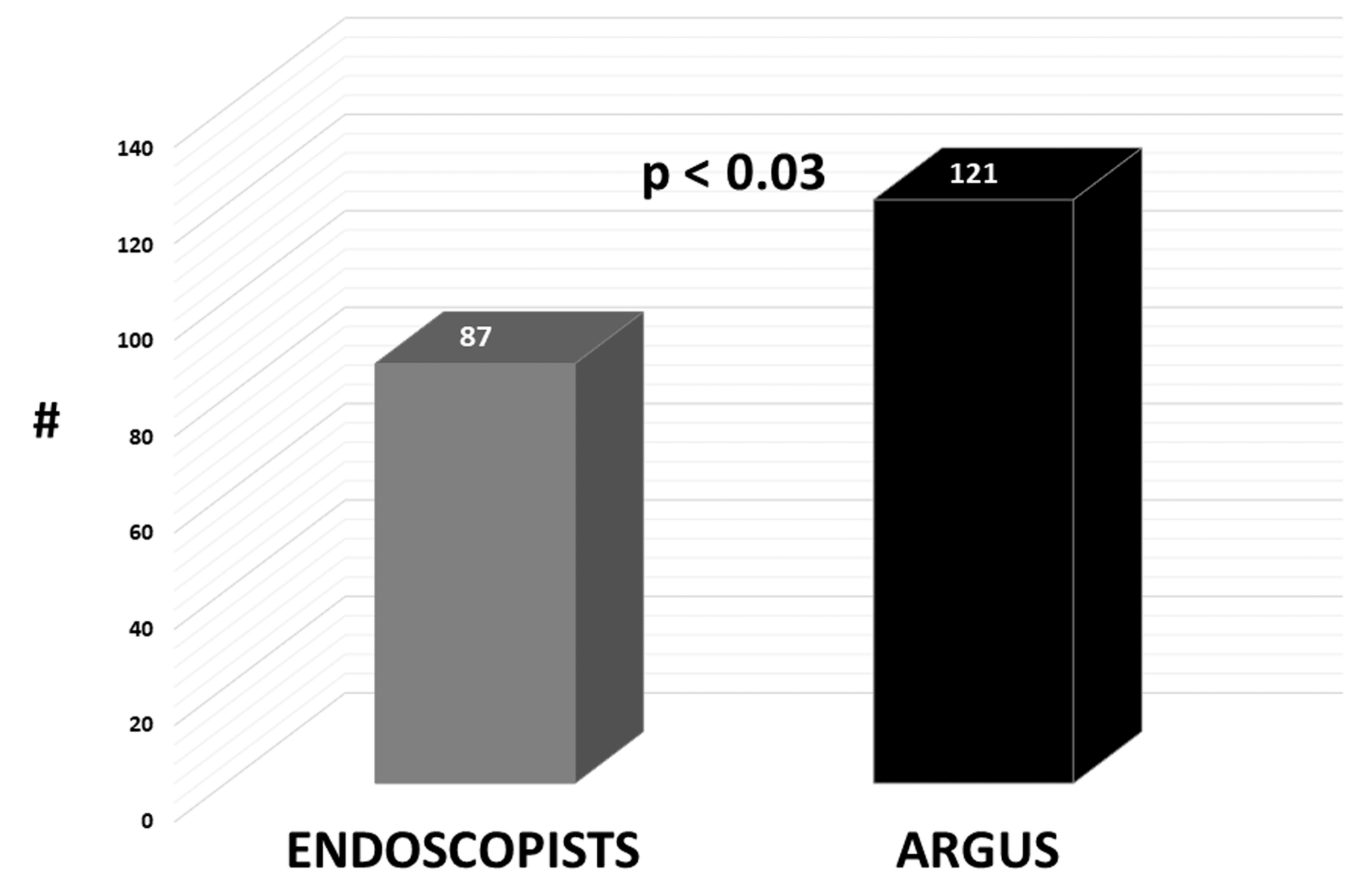
Comparison of appropriate surveillance intervals: endoscopists versus Argus.

Based on polyp size, endoscopists’ surveillance recommendations were significantly more often inappropriate compared to Argus (34 vs 0) (p < 0.03) (Figure 6).

 The interval for the next colonoscopy was too short for 27 endoscopists (22%) and too long for seven endoscopists (6%).

## Discussion

Polyp size estimation is an important aspect of colonoscopy, as the size of a polyp can help determine risk stratification, the appropriate management technique for polypectomy, and follow-up intervals for surveillance colonoscopy. The current standard of practice in colonoscopy is that the endoscopist estimates polyp size based on visual inspection and comparison of the polyp size to the size of the biopsy forceps or snare used for polypectomy [12]. However, endoscopists’ estimation of polyp size can be subjective and has been shown to have significant variability and inaccuracy [13-16]. The reported rate of inaccurate estimation of polyp size ranges from 47-57%, with a subjective rate of polyp size overestimation greater than 70% [14-15]. Inaccurate judgment of polyp size may result in underestimation or overestimation of surveillance colonoscopy that deviates from practice guidelines, negatively impacting patient outcomes and potentially increasing healthcare costs.

The results of this study indicate that AI technology may be more accurate than gastroenterologists in estimating polyp size during colonoscopy. Our results demonstrate that experienced gastroenterologists have a higher proportion of inaccurate measurements of polyp size compared to AI technology. There is an increased tendency among gastroenterologists to overestimate polyp size, which resulted in recommending surveillance colonoscopy intervals shorter than practice guidelines suggest. Similar results are shown by Otero-Regino W et al., where experts tended to overestimate polyp size, such as polyps with a diameter of 7 mm appearing to be 12 mm, those with an 8 mm diameter appearing to measure 15 mm, and those with a 9 mm diameter appearing to be 18 mm [17]. The endoscopists’ overestimation or underestimation of polyp size may be impacted by the quality of bowel preparation, image magnification, and polyp location. Inaccurate measurements of polyp size may be influenced by the polyp being in a difficult location (usually behind folds) or in an area of poor bowel preparation. In addition to maintaining quality metrics of colonoscopy, the incorporation of innovative technology into the detection and measurement of colon polyp size may improve the accuracy and appropriateness of surveillance intervals.

Our findings suggest that the utilization of AI technology during colonoscopy provides a significantly more accurate estimation of polyp size than experienced gastroenterologists. AI is a useful tool for endoscopists, providing real-time correct polyp sizes that assist in deciding the correct interval for surveillance colonoscopy. Despite the initial cost of purchasing AI technology, its long-term utilization in colonoscopy could lead to better resource utilization and significant cost reduction by minimizing both overutilization and underutilization of surveillance colonoscopy [18-20]. The impact of polyp size on the surveillance interval and the finding that gastroenterologists were too short in their estimation 22% of the time suggest that future studies could reveal substantial cost savings

Our study has many strengths. First, this is a novel AI system that is being tested. Second, two separate accuracy measurements were utilized, both showing similar results. Third, by using a phantom colon model, the gold standard of measurement was achieved using a caliper for each polyp. Lastly, the potential impact of incorporating AI technology in accurately estimating polyp size and surveillance colonoscopy intervals is unique and could potentially shift the paradigm of clinical practice guidelines in the future. The widespread application of these findings could offer a cost-effective approach to colorectal cancer surveillance.

As with any study, limitations do exist. First, our study had a small sample size with only 11 polyp videos for 11 endoscopists. However, a type II error was not apparent, given that the outcomes reached statistical significance. Also, despite our study's small sample size and lack of power and external validity, the generalizability of our findings to a larger population is noteworthy. Second, our study provides limited baseline data on the differences between gastroenterologists' and AI's objective assessments of polyp size for polyps ranging from 2.5 to 15 mm. However, further studies are necessary to evaluate AI performance in assessing larger polyps (>15 mm), different polyp morphologies (pedunculated, flat, depressed), and polyps involving more than two folds of the colon. Third, our study’s assessment of surveillance colonoscopy intervals is limited to polyp size measurement only. In the real world, polyp pathology is also essential in risk stratification and determining surveillance colonoscopy intervals. However, due to the experimental nature of our study, we assigned all created polyps to be tubular adenomas with no high-grade dysplasia. Finally, our study was a survey study using videos assessed by volunteers.

## Conclusions

The impact of AI on the polyp detection rate during colonoscopy may improve patient outcomes and reduce healthcare costs associated with missed polyps and unnecessary follow-up procedures due to variability in determining polyp size among different endoscopists. However, it is important to note that AI should be used in conjunction with clinical expertise and judgment, and not as a replacement for human observation and decision-making. Future prospective clinical trials involving multiple centers are necessary to validate the value of AI in estimating polyp size and determining surveillance colonoscopy intervals compared to experienced gastroenterologists' objective assessments.
